# Extracorporeal Cardiac Shock Waves Therapy Improves the Function of Endothelial Progenitor Cells After Hypoxia Injury *via* Activating PI3K/Akt/eNOS Signal Pathway

**DOI:** 10.3389/fcvm.2021.747497

**Published:** 2021-10-11

**Authors:** Mingqiang Wang, Dan Yang, Zhao Hu, Yunke Shi, Yiming Ma, Xingyu Cao, Tao Guo, Hongbo Cai, Hongyan Cai

**Affiliations:** ^1^Department of Cardiology, The First Affiliated Hospital of Kunming Medical University, Kunming, China; ^2^Department of Cardiology, Yunnan Fuwai Cardiovascular Hospital, Kunming, China; ^3^Department of Vascular Surgery, The First Affiliated Hospital of Kunming Medical University, Kunming, China

**Keywords:** extracorporeal cardiac shock waves, endothelial progenitor cells, cell function, PI3K/Akt/eNOS signaling pathways, hypoxia injury, nitric oxide (NO)

## Abstract

**Background:** Extracorporeal cardiac shock waves (ECSW) have great potential in the treatment of coronary heart disease. Endothelial progenitor cells (EPCs) are a class of pluripotent progenitor cells derived from bone marrow or peripheral blood, which have the capacity to migrate to ischemic myocardium and differentiate into mature endothelial cells and play an important role in neovascularization and endothelial repair. In this study, we investigated whether ECSW therapy can improve EPCs dysfunction and apoptosis induced by hypoxia and explored the underlying mechanisms.

**Methods:** EPCs were separated from ApoE gene knockout rat bone marrow and identified using flow cytometry and fluorescence staining. EPCs were used to produce *in vitro* hypoxia-injury models which were then divided into six groups: Control, Hypoxia, Hypoxia + ECSW, Hypoxia + LY294002 + ECSW, Hypoxia + MK-2206 + ECSW, and Hypoxia + L-NAME + ECSW. EPCs from the Control, Hypoxia, and Hypoxia + ECSW groups were used in mRNA sequencing reactions. mRNA and protein expression levels were analyzed using qRT-PCR and western blot analysis, respectively. Proliferation, apoptosis, adhesion, migration, and angiogenesis were measured using CCK-8, flow cytometry, gelatin, transwell, and tube formation, respectively. Nitric oxide (NO) levels were measured using an NO assay kit.

**Results:** Kyoto encyclopedia of genes and genomes (KEGG) enrichment analysis showed that differentially expressed genes were enriched in cancer signaling, PI3K-Akt signaling, and Rap1 signaling pathways. We selected differentially expressed genes in the PI3K-Akt signaling pathway and verified them using a series of experiments. The results showed that ECSW therapy (500 shots at 0.09 mJ/mm^2^) significantly improved proliferation, adhesion, migration, and tube formation abilities of EPCs following hypoxic injury, accompanied by upregulation of p-PI3K, p-Akt, p-eNOS, Bcl-2 protein and NO, PI3K, and Akt mRNA expression, and downregulation of Bax and Caspase3 protein expression. All these effects of ECSW were eliminated using inhibitors specific to PI3K (LY294002), Akt (MK-2206), and eNOS (L-NAME).

**Conclusion:** ECSW exerted a strong repaired effect on EPCs suffering inhibited hypoxia injury by inhibiting cell apoptosis and promoting angiogenesis, mainly through activating the PI3K/Akt/eNOS signaling pathway, which provide new evidence for ECSW therapy in CHD.

## Introduction

Coronary heart disease (CHD) is the leading cause of death in adults worldwide (1, 2). Although existing drug therapy, percutaneous coronary intervention (PCI) and coronary artery bypass grafting (CABG) have greatly improved the symptoms and prognosis of most patients with CHD. However, there is still a need to prevent myocardial ischemia and improve the quality of life of patients who cannot tolerate surgery or who continue to experience angina pectoris after receiving optimal drug or surgical treatment (3, 4). The recently developed extracorporeal cardiac shock waves (ECSW) therapy is a new non-invasive treatment for CHD. Its safety and efficacy have been demonstrated in animal models and in clinical trials (5–8). Current research suggests that, through tissue cavitation, ECSW produce a series of biochemical effects, including shear stress on cell membranes (9), increased endothelial nitric oxide synthase (eNOS) and nitric oxide (NO) synthesis, and upregulation of vascular endothelial growth factor (VEGF), which attenuates cell apoptosis, inflammatory responses and induces angiogenesis (10–12). Another potential cellular mechanism may involve ECSW inducing endothelial progenitor cells (EPCs) to home to ischemic sites, exerting pro-angiogenesis, anti-oxidative stress, anti-inflammation, and anti-fibrosis effects (13–15). However, the exact mechanism through which ECSW stimulate angiogenesis and improves myocardial function is still unknown.

EPCs are a class of pluripotent progenitor cells derived from bone marrow or peripheral blood. Studies have shown that EPCs can migrate from the bone marrow to the ischemic myocardium, where they differentiate into mature endothelial cells and participate in the repair of vascular endothelium and neovascularization in the injured site (16–18). Therefore, EPCs are ideal candidates for angiogenesis. Recent studies have found that the number and function of EPCs are impaired in patients with ischemic heart diseases such as coronary heart disease, indicating that CHD not only directly weakens the function of vascular endothelia but also retards the endothelial repair process mediated by EPCs (19, 20). Consequently, ECSW treatment to improve EPCs function in patients with CHD may be an effective new strategy for preventing and treating ischemic heart disease.

In the past, it has been found that the combined treatment of ECSW with intracoronary administration of autologous bone marrow-derived EPCs showed convincing results in improving left ventricular ejection fraction in patients with chronic heart failure (21). Another evidence has shown that ECSW therapy can increase recruitment and homing of endogenous EPCs derived from autologous bone marrow to the damaged ischemic myocardium, promote angiogenesis, and improve myocardial ischemia (22). These results suggest that ECSW have great potential in activating EPCs *in vivo*, but the mechanism of ECSW to improve EPC function is still poorly understood. In this study, we focused on the effects of ECSW on EPCs under hypoxic-ischemic microenvironment, then used bioinformatics analyses and relevant pathway validation to investigate underlying mechanism, in order to gain further understanding of ECSW therapy for CHD.

## Materials and Methods

### Isolation and Culturing of EPCs

All procedures and protocols involving animals were approved by the Institutional Animal Care and Use Committee of the First Affiliated Hospital of Kunming Medical University (Yunnan, China) and performed in accordance with the Guide for the Care and Use of Laboratory Animals (Animal Ethics NO. Kmmu2021109). The main pathological basis of coronary heart disease (CHD) is the development of atherosclerosis. It has been found that ApoE gene knockout rats could spontaneously form hyperlipidemia and atherosclerosis in normal diet, which is similar to human atherosclerotic pathological process (23). On this basis, EPCs were isolated from ApoE gene to knockout rat bone marrow for closer to the pathological model of atherosclerosis in CHD. EPCs were isolated from 4-week-old ApoE gene knockout rats bone marrow *via* density-gradient centrifugation using Ficoll separating solution (Solarbio, Beijing, China). The EPCs were resuspended in Endothelial Cell Growth Medium (EGM-2 MV) (Lonza, Walkersville, Maryland, USA) containing 10% fetal bovine serum and cultivated at 37°C and 5% CO_2_. After 3 days of culturing, non-adherent cells were removed by washing with phosphate buffered saline (PBS) and new medium was added every 3–4 days. The 2nd−6th generation cells were detached using trypsin and collected for further experiments.

### Identification of EPCs

Following 14 days of culturing, the cells were incubated with 20 μg/ml Dil-acetylated low-density lipoprotein (Dil-Ac-LDL; Maokang Biotechnology, Shanghai, China) at 37°C and 5% CO_2_ for 4 h and then fixed at room temperature for 20 min using 4% paraformaldehyde before being incubated with 10 μg/ml FITC labeled Mlex Europaeus Agglutinin I (FITC-UEA-I; Maokang Biotechnology, Shanghai, China) at room temperature for 1 h. After staining and mounting with DAPI (Solarbio Biotechnology, Beijing, China), the slides were observed under a fluorescent microscope. Dual-stained cells (positive for Dil-Ac-LDL and FITC-UEA-I) were identified as EPCs. Furthermore, the EPCs marker profile was investigated using flow cytometry. EPCs were collected, digested, and fixed with 4% paraformaldehyde at room temperature for 20 min, then centrifuged at 1,000 rmp for 5 min and washed once with PBS. The cells were blocked at room temperature for 15 min using 5% BSA (Solarbio Biotechnology, Beijing, China), centrifuged at 1,000 rmp for 5 min and incubated overnight at 4°C using 1:100 dilutions of either rabbit anti-CD34 antibody (Bioss Biotechnology, Beijing, China), rabbit anti-CD133 antibody (Bioss Biotechnology, Beijing, China), rabbit anti-CD31 antibody (Bioss Biotechnology, Beijing, China), or rabbit anti-VEGFR2 antibody (Bioss Biotechnology, Beijing, China) to allow hybridization. The cells were then centrifuged at 1,000 rmp for 5 min before being washed once with PBS and incubated with FITC-labeled goat anti-rabbit lgG antibody (Bioss Biotechnology, Beijing, China) at room temperature for 2 h. This was followed by centrifugation at 1,000 rmp for 5 min and wash with PBS. Finally, a cell suspension was prepared in PBS. Flow cytometry was used to detect the percentage of stained EPCs. Relative isotype controls were used as negative controls.

### Experimental Protocol

To simulate the ischemic and hypoxic microenvironment in CHD, EPCs were exposed to hypoxic conditions (1% O_2_, 95% N_2_, 5% CO_2_) and starved with 1% fetal bovine serum in EBM-2 medium for 24 h. Cells incubated under normoxic conditions (95% O_2_, 5% CO_2_) and EGM-2 MV medium for 24 h were used as controls. The EPCs were then divided into six groups: (1) control group; (2) hypoxia group; (3) hypoxia + ECSW group (hypoxia injury model and for extracorporeal cardiac shock waves treatment); (4) hypoxia + LY294002 + ECSW group (hypoxia injury model treated with LY294002 (selleck, Houston, Texas, USA; 100 μM) to block PI3K for 6 h before ECSW treatment); (5) hypoxia + MK-2206 + ECSW group (hypoxia injury model treated with MK-2206 (selleck, Houston, Texas, USA; 20 μM) to block Akt for 6 h before ECSW treatment); (6) hypoxia + L-NAME + ECSW group (hypoxia injury model treated with L-NAME (selleck, Houston, Texas, USA; 1 mM) to block eNOS for 6 h before ECSW treatment). ECSW treatment was measured using the MODULITH SLC instrument (STORZ MEDICAL, Switzerland) with an energy of 0.09 mJ/mm^2^ and a total of 500 shots.

### RNA-seq and Bioinformatics Analysis

For each cell sample, preparation of tagged mRNA sequencing libraries, sequencing, and data analysis were performed by LC Sciences (China). EPCs were cultured and divided into 3 experimental groups (*n* = 3): (1) control, (2) hypoxia, and (3) hypoxia + ECSW. Total RNA was extracted using TRIzol reagent (Invitrogen, CA, USA). The total RNA quantity and purity were analyzed using Bioanalyzer 2100 and RNA 6000 Nano LabChip Kit (Agilent, CA, USA) with RIN number >7.0. Following purification, the mRNA was fragmented into small pieces using divalent cations. The cleaved RNA fragments were then reverse-transcribed to create the final cDNA library following the mRNA-seq sample preparation kit (Illumina, San Diego, USA) protocol. The average insert size for the paired-end libraries was (300 bp ± 50 bp). Paired-end sequencing was performed on an Illumina Hiseq 4000 at LC Sciences, USA, following the vendor's protocol. Prior to assembly, the low-quality reads (reads containing sequencing adaptors, reads containing sequencing primer, and nucleotides with quality scores lower than 20) were removed, leaving clean, paired-end reads.

Sample reads were aligned to the reference genome using HISAT package, which initially removes a portion of the reads based on the quality information accompanying each read and then maps them to the reference genome. HISAT allows multiple alignments per read (up to 20 by default) and a maximum of two mismatches when mapping the reads to the reference. HISAT generates a database of potential splice junctions and confirms these by comparing the previously unmapped reads against the database of putative junctions. The mapped reads from each sample were assembled using StringTie. All transcriptomes from samples were then merged to reconstruct a comprehensive transcriptome using perl scripts. After the final transcriptome was generated, StringTie and edgeR were used to estimate the expression levels of all transcripts. StringTie was used to analyze mRNA expression levels by calculating FPKM. Differentially expressed mRNAs and genes were selected based on log2 (fold change) >1 or log2 (fold change) <-1 and *p* < 0.05).

We performed GO enrichment analysis to predict the functions and mechanisms of mRNA and differentially expressed genes. Significant GO terms were defined as those with *P* < 0.05. In addition, Kyoto encyclopedia of genes and genomes (KEGG) enrichment analysis to predict the signaling pathways in which mRNA may participate was conducted using KEGG Orthology Based Annotation System (KOBAS) v3.0 software. Significant signaling pathways with *P* < 0.05 were included in the analysis.

### Proliferation Assays

Cell Proliferation was evaluated using Cell Counting Kit-8 (CCK-8; Dojindo Co, Japan) assays, following the manufacturer's instructions. EPCs (1 × 10^3^) from each group were cultured in two 96-well plates. Ten microliter of CCK-8 solution was added to each well in one of the 96-well plates and incubated for 2 h at 37°C and 5% CO_2_. The absorbance at 450 nm (OD450) was measured using an enzyme labeling apparatus (Molecular Devices, USA). After 24 h, 10 μl of CCK8 solution was added to each well of the second 96-well plate and incubated for 2 h at 37°C and 5% CO_2_. The absorbance of the solution in each well was measured with an enzyme labeling apparatus at 450 nm (OD450). The differences between 24 and 0 h absorbance values were used to determine EPCs proliferation ability in each group.

### Apoptosis Assays

EPCs apoptosis was detected using Annexin V, FITC Apoptosis Detection Kit (Dojindo Co, Japan). EPCs from each group were digested and collected, washed twice with PBS, and resuspended in 500 μl of 1 × Annexin V Binding Solution. Hundred microliter cell suspension was added to new tubes, combined with 5 μl of Annexin V-FITC Binding and PI Solution for 15 min incubated at room temperature in the dark. Then, 400 μl of 1 × Annexin V Binding Solution was added and the mixture analyzed using flow cytometry within 1 h.

### Adhesion Assay

2 × 10^4^ cells from each EPCs group were inoculated in each well of a 6-well plate coated with 1% gelatin and incubated for 1 h at 37°C and 5% CO_2_. The non-adherent cells were sucked out and washed three times with PBS. Five random regions were taken from each group and adherence in each group was counted under the microscope. The mean number of adherent cells was used to determine the adhesion ability of EPCs in each group.

### Transwell Assay

EPCs in each group were collected, resuspended in EBM-2 medium, and 2 × 10^4^ of the cells were added to each well in the upper chambers of transwell plates (Corning, New York, USA). EGM-2 MV medium containing 10% fetal bovine serum was added to the lower chambers to promote EPCs migration. The transwell plate was incubated for 24 h at 37°C and 5% CO_2_. The transwell chamber was carefully removed, and the cells that had not passed through the membrane and the medium were washed out with PBS. The remaining cells were fixed with 4% paraformaldehyde for 20 min. The transwell chamber was stained with 1% crystal violet solution (Solarbio, Beijing, China) for 20 min, washed with PBS and the cells that had not passed through the membrane were removed with a cotton swab. The non-cellular inoculation side was photographed under an upright microscope.

### Tube Formation Assay

EPCs tube formation assay was evaluated using Corning Matrigel Basement Membrane Matrix (Corning, New York, USA). The Matrigel matrix was melted overnight at 4°C and added into a pre-cooled 96-well plate (50 μl/well), then incubated at 37°C for 1 h to allow coagulation. EPCs in each group were inoculated into wells (2 × 10^4^ cells/well) on top of the solidified Matrigel matrix. Hundred microliter of EGM-2 MV medium with 10% fetal bovine serum was added and the plates incubated for 8 h in an incubator. Tube formation was quantified by counting sprouting microcapillary-like structures with lengths four times their width using an inverted microscope.

### Quantitative Real-Time Polymerase Chain Reaction

The levels of PI3K and Akt in EPCs from each group were determined using real-time reverse transcription polymerase chain reaction. The total RNA in each group was extracted using TRIzol (Thermo Fisher Scientific, Waltham, Massachusetts, USA). The extracted total RNA was purified and converted into cDNA using the TaKaRa PrimeScript™ RT reagent Kit with gDNA Eraser (TAKARA, Tokyo, Japan). The Life Technology company's applied biosystems QuantStudio 6 Flex real-time fluorescence PCR instrument and the TaKaRa TB Green Primix Ex Taq™ II Kit (TAKARA, Tokyo, Japan) were used for amplification. Relative quantities of PI3K and Akt were normalized to that of the housekeeping gene, GAPDH, and the relative changes in the expression of target genes were evaluated using the 2^−ΔΔCT^ method. The primers used for PI3K, Akt, and GAPDH are as follows: PI3K (F) 5′-CTGTGCCTTCTGCCTTACGGTTG-3′, (R) 5′-GCAATCGTCGTGGCGTCCTTC-3′; Akt (F) 5′-GGCAGGAGGAGGAGACGATGG-3′, (R) 5′-TTCATGGTCACACGGTGCTTGG-3′; GAPDH (F) 5′-ACGGCAAGTTCAACGGCACAG-3′, (R) 5′-CGACATACTCAGCACCAGCATCAC-3′.

### Western Blot Analysis

EPCs from each group were lysed using RIPA lysis buffer containing 1% protease and phosphatase inhibitors (Beyotime Biotechnology, Shanghai, China). The protein concentrations in each group were measured using BCA protein concentration assay kit (Beyotime Biotechnology, Shanghai, China). A total of 30 μg protein from each group was separated on 8% sodium dodecyl sulfate (SDS) polyacrylamide gels (Beyotime Biotechnology, Shanghai, China) and transferred onto PVDF membrane (Millipore, USA). The membrane was incubated with 5% non-fat milk at room temperature for 4 h to block non-specific binding, and incubated overnight at 4°C with appropriately diluted of primary antibodies. The antibodies included rabbit PI3K antibody (1:1,000, AF6241, Affinity), rabbit phospho-PI3K antibody (1:1,000, AF3242, Affinity), rabbit Akt antibody (1:1,000, #4691, Cell Signaling Technology), rabbit phospho-Akt antibody (1:1,000, #4060, Cell Signaling Technology), rabbit eNOS antibody (1:1,000, #32027, Cell Signaling Technology), rabbit phospho-eNOS antibody (1:250, #07-428-I, Millipore), rabbit Bax antibody (1:1,000, #2772, Cell Signaling Technology), rabbit Bcl-2 antibody (1:1,000, AF6139, Affinity), rabbit Cleaved Caspase-3 antibody (1:1,000, #9664, Cell Signaling Technology), and rabbit β-actin antibody (1:1,000, #4970, Cell Signaling Technology). After incubation, it was washed three times with TBST and incubated with horseradish-peroxidase-conjugated secondary antibodies (1:5,000, Cat. #S0001, Affinity) for 2 h at room temperature. The membranes were visualized using the Immobilon Western Chemiluminescent HRP Substrate (ECL, Millipore, USA), and the Gel-Pro Analyzer software was used to quantify the gray scale data of protein bands. β-actin was used to control for unequal loading.

### Measurement of Nitric Oxide Levels

Nitric Oxide (NO) levels in the cells were measured using the NO assay kit (Solarbio, Beijing, China) according to the manufacturer's instructions. Cells in each group were digested and collected, 800 μl extract added, and the cells crushed using an ice bath ultrasound (power 300 W, ultrasound 3S, interval 7s, total time 3 min). The cells were centrifuged at 4°C for 15 min at 12,000 rmp. The precipitate was discarded and the supernatant measured. Hundred microliter of the supernatant was added to new tubes, 20 μl Reagent 1 added, the solution vortexed to mix. The solution was placed in a water bath for 60 min at 37°C, 20 μl Reagent 2 added and vortexed to mix, reacted for 5 min at room temperature. The mixture was then centrifuged at 3,500 rmp for 10 min. Hundred microliter of supernatant was transferred to the 96-well plate, and 100 μl of chromogenic solution added, mixed, and incubated for 10 min at room temperature. Absorbance of the solution was measured with an enzyme labeling apparatus (Molecular Devices, USA) at 550 nm (OD550).

### Statistical Analysis

All results are expressed as means ± SD of at least three repeated experiments and were analyzed using Graph Pad Prism 8.0 software (GraphPad Software, Inc., USA). One-way ANOVA followed by the Tukey's *post-hoc* test was conducted among multiple groups comparisons, and unpaired Student *t*-test was used for two-group comparisons. *P* < 0.05 was regarded statistically significant.

## Results

### Characterization of EPCs

**Figure 1:**
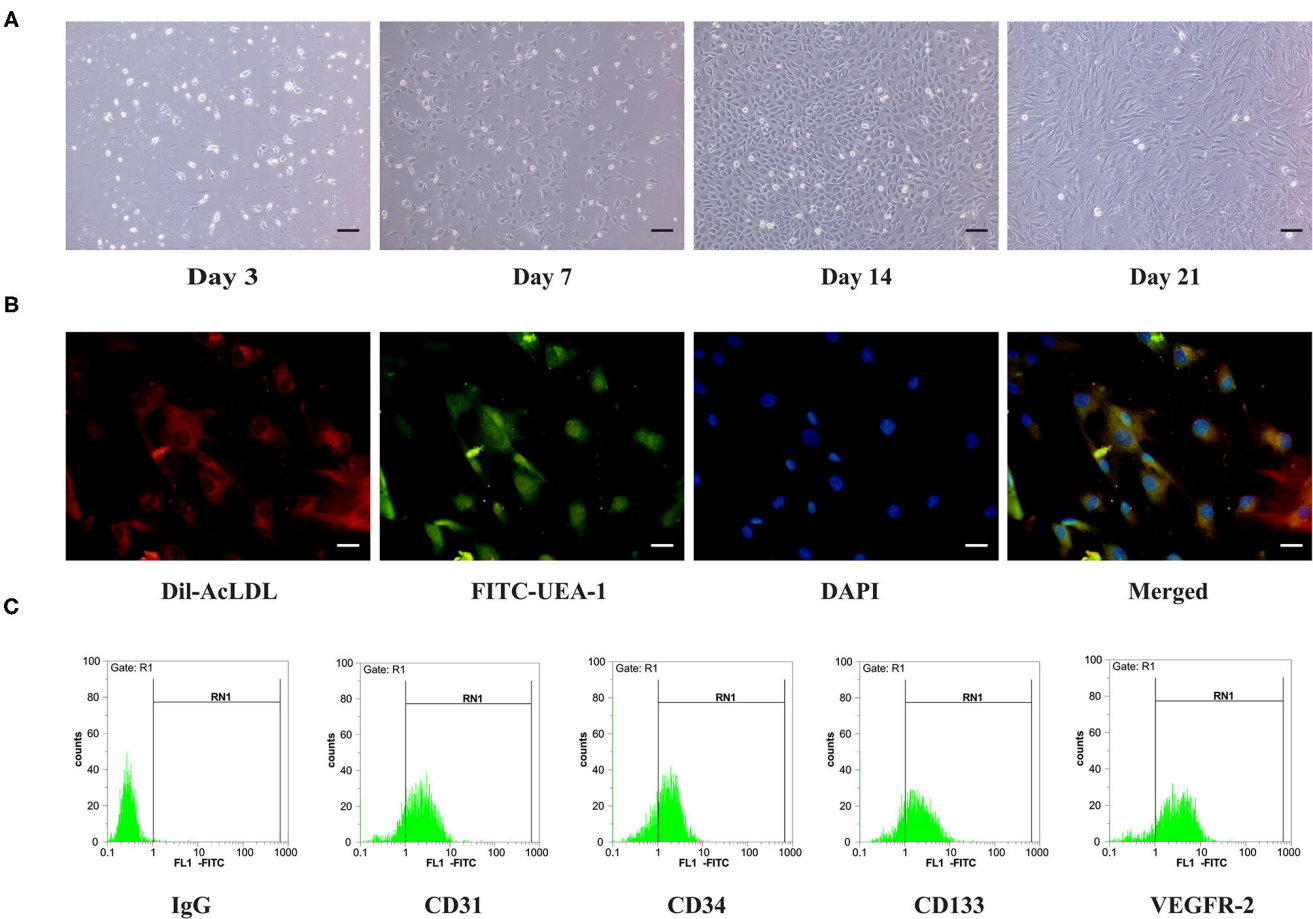
Characterization of ApoE gene knockout rat bone marrow-derived EPCs. **(A)** On Day 3, Day 7, Day 14, and Day 21 of culture, the morphology of EPCs is observed under a microscope. Scale bar =100 μm. **(B)** EPCs taking up DiI-Ac-LDL could be visualized (red), immunofluorescence staining of EPCs positive for FITC-UEA-1 (green), nucleus (blue) was stained by DAPI. Scale bar =50 μm. **(C)** Cell surface markers (CD31, CD34, CD133, and VEGFR-2) of EPCs were investigated using flow cytometry.

Cells were cultured in medium containing 10% FBS/EGM-2MV after isolation from 4-week-old ApoE gene knockout rat bone marrow. On day 3, non-adherent cells were removed and adherent, oval, or short spindle cells were observed. On day 7, some cells changed their appearance and formed colonies. On day 14, the cells showed typical colony growth and exhibited a cobblestone-like morphology. On day 21, the cells exhibited a fusiform shape, with a morphology similar to that of endothelial cells (Figure 1A).

EPCs are characterized by their ability to take up Ac-LDL and bind to UEA-I. In our experiment, after coculturing with Dil-Ac-LDL and UEA-I, positive staining in these cells was observed for Dil-Ac-LDL and UEA-I using fluorescence microscopy (Figure 1B). Furthermore, the surface antigens of these cells were investigated using flow cytometry. Because it is difficult to identify EPCs by staining using a single surface marker, staining with a combination of surface markers (e.g., CD31, CD34, CD133, and VEGFR2) was necessary. Flow cytometry showed that most of the cells were positive for CD31, CD34, CD133, and VEGFR2 (Figure 1C). These basic characterizations indicate that EPCs were successfully isolated from ApoE gene knockout rat bone marrow.

### Gene Ontology Analysis and KEGG Pathway Annotation of Differentially Expressed Genes

**Figure 2:**
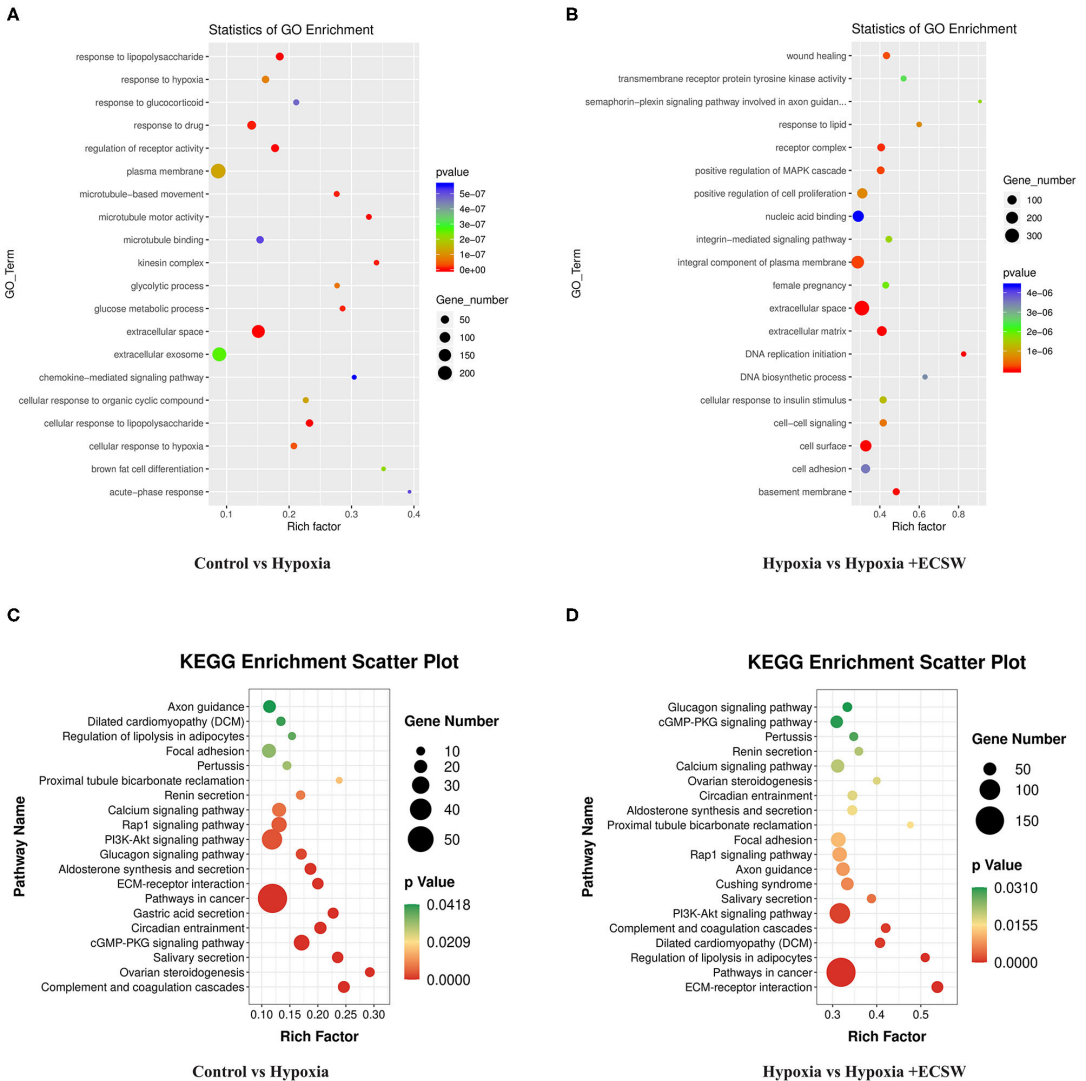
Bioinformatic analysis of differentially expressed genes (DEGs). **(A,B)** Functional annotation of DEGs based on Gene ontology analysis. **(A)** Hypoxia group EPCs represent the treatment group, Control group EPCs are the negative control. **(B)** Hypoxia + ECSW group EPCs are the treatment group, Hypoxia group EPCs are the negative control. **(C,D)** Functional annotation of differentially expressed mRNAs based on KEGG pathway analysis. **(C)** Hypoxia group EPCs are the treatment group, Control group EPCs are the negative control. **(D)** Hypoxia + ECSW group EPCs are the treatment group, Hypoxia group EPCs are the negative control.

The differentially expressed mRNAs and the potential signaling pathways that are involved were analyzed using bioinformatics methods. Gene ontology (GO) analysis revealed that, compared with the control group, important GO terms were significantly enriched in the positive regulation of extracellular space, response to drug, and plasma membrane in the hypoxia group. The 20 most common GO categories that were enriched are shown in Figure 2A. Compared with the hypoxia group, some important GO terms were significantly enriched in the positive regulation of extracellular space, cell surface, and integral component of plasma membrane in the hypoxia +ECSW group. The 20 most common GO categories that were enriched are shown in Figure 2B. To distinguish the biological pathways that became active in EPCs of the hypoxia and hypoxia +ECSW groups, we investigated the differentially expressed mRNAs using term enrichment analysis to identify their possible targets using the KEGG annotation. The results showed that several important pathways were enriched in cancer signaling, PI3K-Akt signaling, and Rap1 signaling pathways. As shown in Figures 2C,D, the 20 most common pathways were enriched in EPCs from both the hypoxia and hypoxia +ECSW groups.

### ECSW Inhibit Apoptosis and Promotes the Proliferation of EPCs Following Hypoxic Injury by Activating the PI3K/Akt/eNOS Signaling Pathway

**Figure 3:**
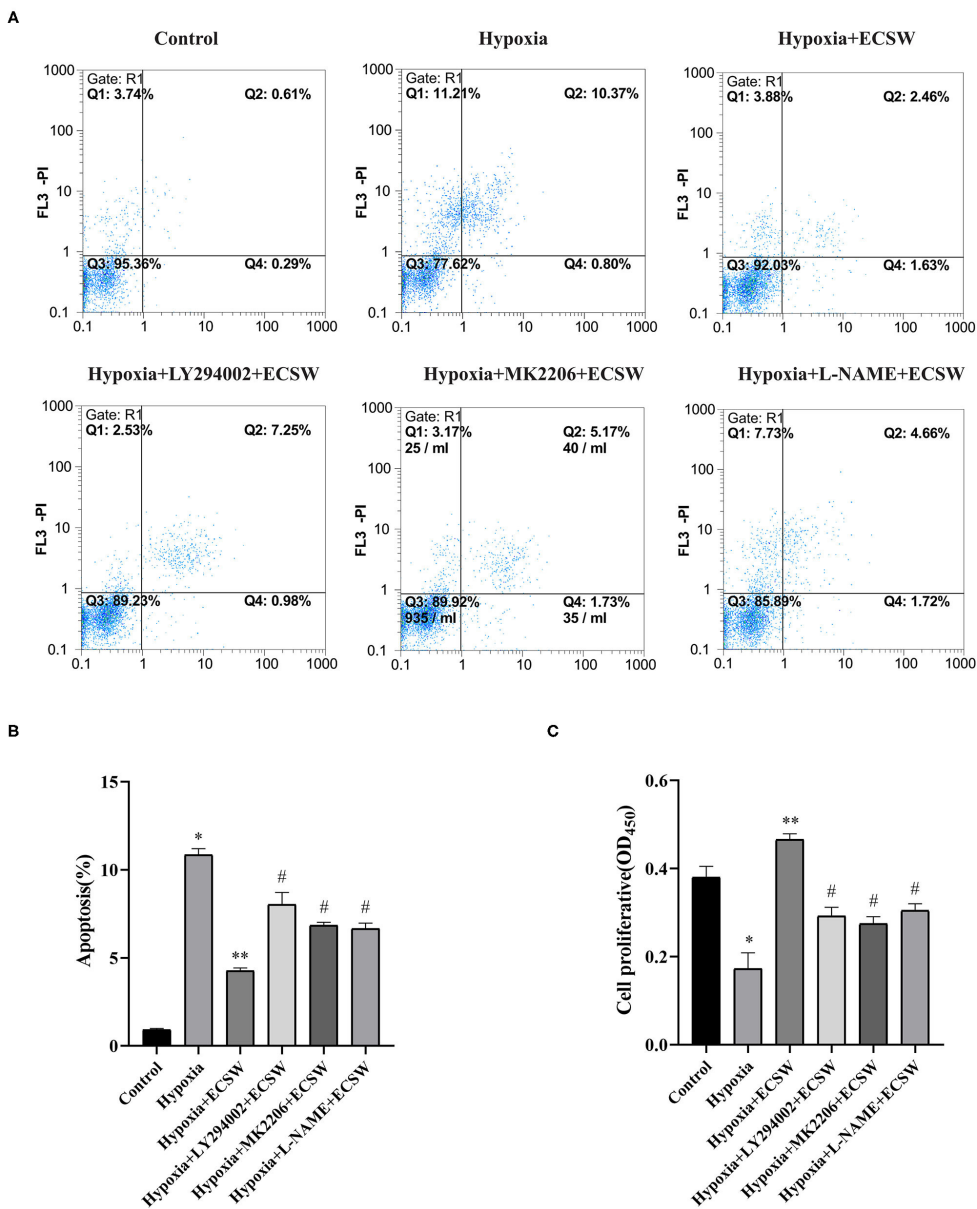
Extracorporeal cardiac shock waves (ECSW) inhibit apoptosis and promotes the proliferation of EPCs after hypoxic injury by activating PI3K/Akt/eNOS signaling pathway. **(A)** Representative images of EPC apoptosis in each group were detected using flow cytometry. **(B,C)** Quantitative analysis of the apoptosis of EPCs and the proliferation of EPCs in each group, respectively. Data are presented as mean ± SD, *N* = 3. *P < 0.05 vs. group control, **P < 0.05 vs. group hypoxia, ^#^*P* < 0.05 vs. group hypoxia + ECSW.

The cell apoptosis assay using Annexin V-FITC and PI double staining (Figures 3A,B) showed that the percentage of apoptotic EPCs was significantly increased by hypoxic challenge (*P* < 0.05). After ECSW treatment, the apoptosis index was significantly lower than that in the hypoxia group (*P* < 0.05). The proliferation of EPCs in each group was detected using the CCK-8 assay. As shown in Figure 3C, hypoxia induced a significant decrease in cell proliferation compared with control cells (*P* < 0.05). ECSW treatment resulted in a significant increase in cell proliferation compared with the hypoxia group (*P* < 0.05). These results revealed that ECSW protected EPCs against hypoxic injury. However, pretreatment with the PI3K inhibitor, LY294002, the Akt inhibitor, MK2206, and the eNOS inhibitor, L-NAME, significantly attenuated the effects of ECSW in EPCs exposed to hypoxic conditions (*P* < 0.05).

### ECSW Promote Adhesive, Migratory and Tube Formation Capacities of EPCs Following Hypoxic Injury by Activating the PI3K/Akt/eNOS Signaling Pathway

**Figure 4:**
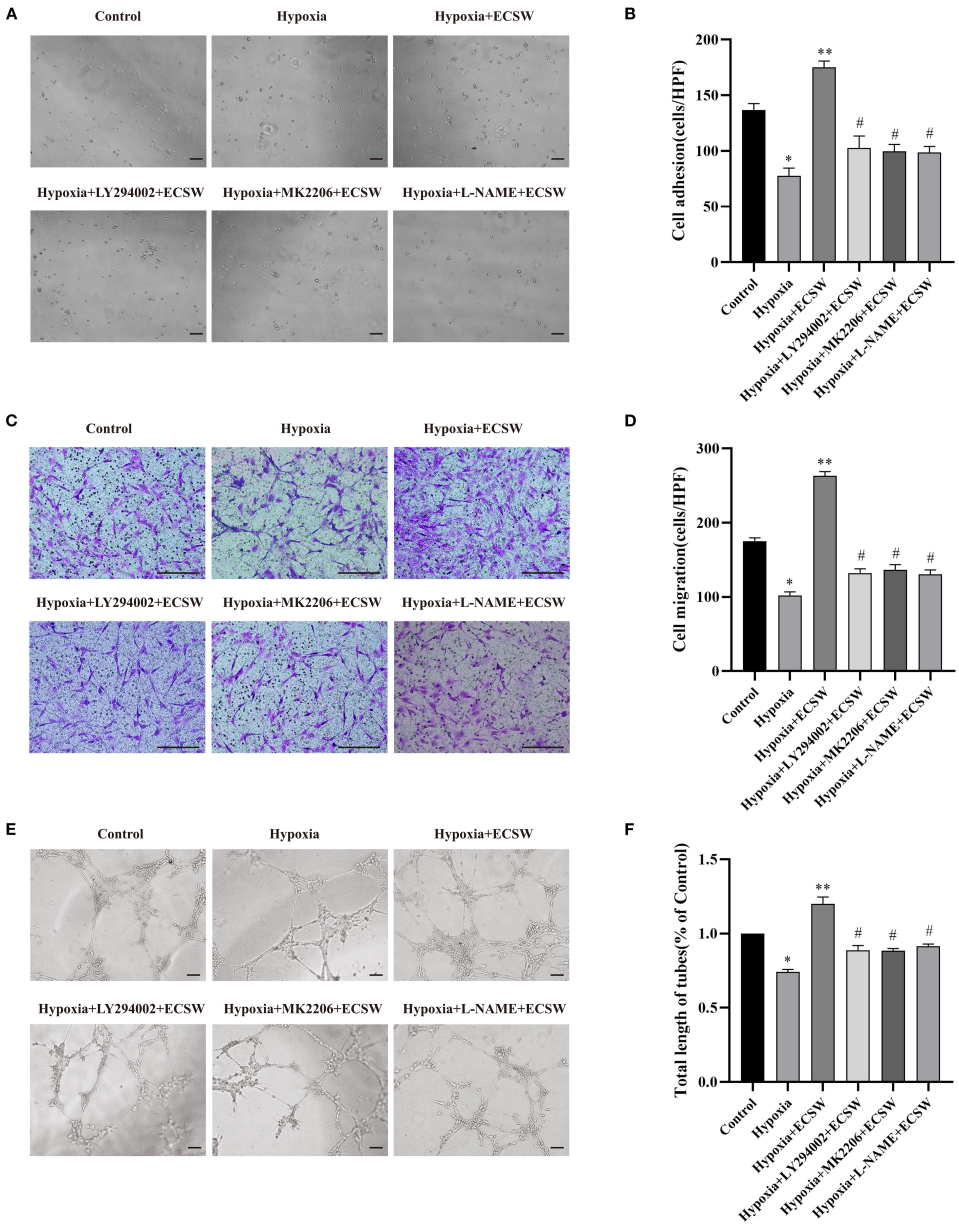
ECSW improve the adhesive, migratory, and tube formation capacity of EPCs after hypoxic injury by activating PI3K/Akt/eNOS signaling pathway. **(A,C,E)** Representative images of EPCs adhesive, migratory, and tube formation in each group under a microscope. **(A,E)** Scale bar = 100 μm; **(C)** Scale bar = 200 μm. **(B,D,F)** Quantitative analysis of the adhesive, migratory, and tube formation of EPCs in each group. Data are presented as mean ± SD, *N* = 3. *P < 0.05 vs. group control, **P < 0.05 vs. group hypoxia, #*P* < 0.05 vs. group hypoxia + ECSW.

Cells adhering onto 1% gelatin-coated 6-well plates were quantified using microscopy and showed that hypoxia challenge induced a significant decrease in the number of EPCs compared with control cells (*P* < 0.05, Figures 4A,B). This impaired adhesion of EPCs exposed to hypoxia was improved following treatment with ECSW (*P* < 0.05, Figures 4A,B). In addition, pretreatment with the PI3K inhibitor, LY294002, the Akt inhibitor, MK2206, and the eNOS inhibitor, L-NAME, inhibited this effect of ECSW in EPCs exposed to hypoxia (*P* < 0.05, Figures 4A,B).

The *in vitro* migratory ability of EPCs was assessed as the ability of EPCs to invade the lower side of the transwell chamber. As shown in Figures 4C,D, the number of successfully migrated cells decreased when exposed to hypoxic conditions compared with control cells (*P* < 0.05). ECSW treatment had a beneficial effect on the migratory ability of EPCs exposed to hypoxic conditions (*P* < 0.05). However, pretreatment with the PI3K inhibitor, LY294002, the Akt inhibitor, MK2206, and the eNOS inhibitor, L-NAME, inhibited this effect of ECSW in EPCs exposed to hypoxia (*P* < 0.05).

EPCs tube formation was detected using a Matrigel assay, and angiogenesis was expressed based on tube length. As shown in Figures 4E,F, the angiogenic ability of EPCs significantly decreased when exposed to hypoxia compared with control cells (*P* < 0.05). The capillary-like vascular tube network became denser following ECSW treatment (*P* < 0.05). In addition, pretreatment with the PI3K inhibitor, LY294002, the Akt inhibitor, MK2206, and the eNOS inhibitor, L-NAME, inhibited this effect of ECSW in EPCs exposed to hypoxia (*P* < 0.05).

### ECSW Activate the EPCs PI3K/Akt/eNOS Signaling Pathway Following Hypoxic Injury

**Figure 5:**
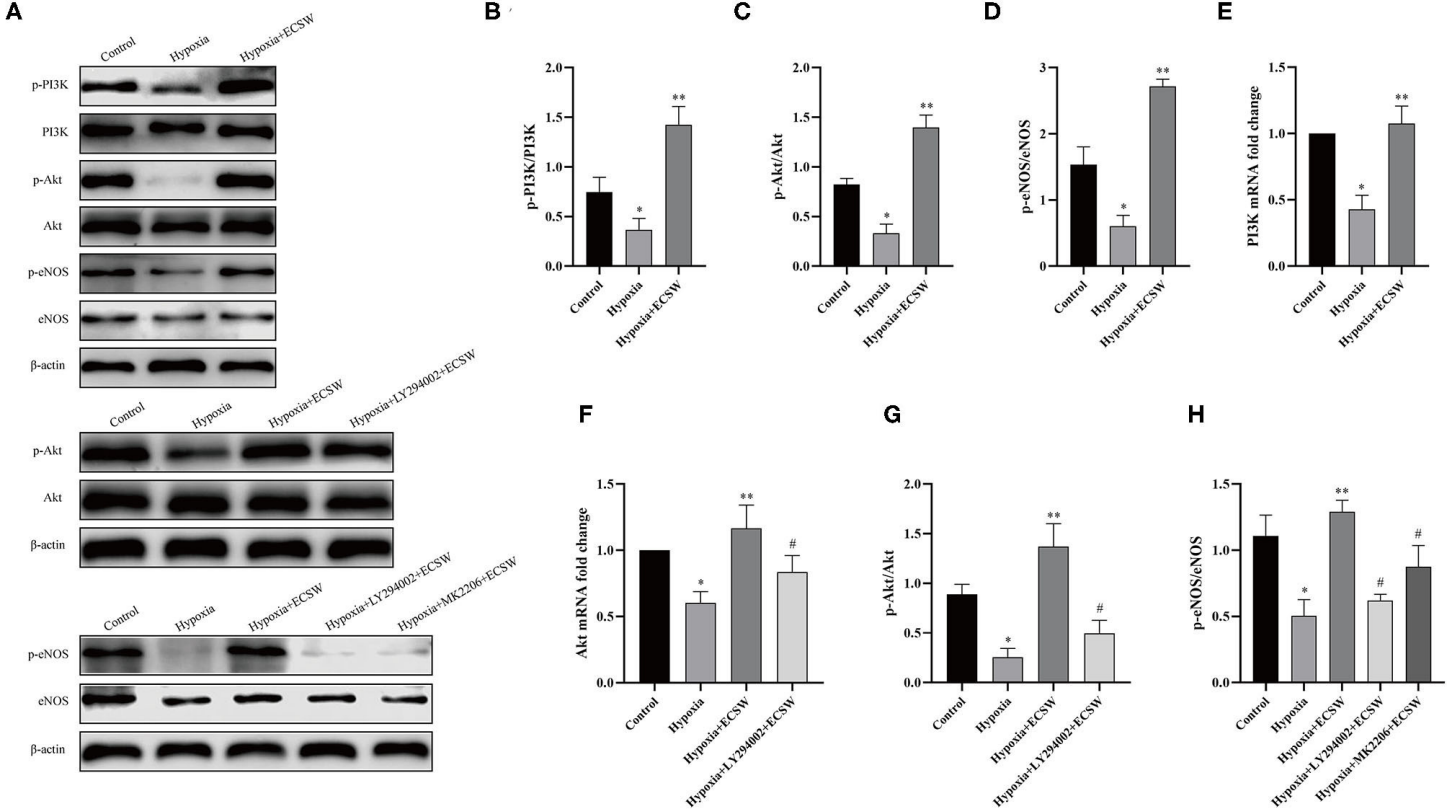
ECSW improve PI3K/Akt/eNOS expression levels of EPCs after hypoxic injury. **(A)** The expression of p-PI3K, PI3K, p-Akt, Akt, p-eNOS, eNOS, and β-actin proteins in EPCs in each group. **(B–D,G,H)** Quantification analysis of p-PI3K, p-Akt, and p-eNOS proteins expression levels based on Western blotting analysis. **(E,F)** Quantitative analysis of mRNA levels of PI3K and Akt of EPCs in each group. Data are presented as mean ± SD, *N* = 3. *P < 0.05 vs. group control, **P < 0.05 vs. group hypoxia, ^#^*P* < 0.05 vs. group hypoxia + ECSW.

To assess the mechanism underlying the injury of EPCs that is induced by hypoxia and the protective effect of ECSW on EPCs, PI3K/Akt/eNOS signaling was detected *via* western blotting and RT-PCR. As shown in Figures 5A–D, the expression of p-PI3K, p-Akt, and p-eNOS in EPCs decreased after exposure to hypoxic conditions for 24 h (*P* < 0.05), and the expression of PI3K, Akt, and eNOS proteins showed no significant changes. ECSW treatment increased the expression of p-PI3K, p-Akt, and p-eNOS (*P* < 0.05), but the levels of PI3K, Akt, and eNOS proteins showed no significant changes in the EPCs exposed to hypoxia. However, pretreatment with the PI3K inhibitor, LY294002, and the Akt inhibitor, MK-2206, inhibited this effect of ECSW in EPCs exposed to hypoxia (*P* < 0.05) (Figures 5G,H). As shown in Figures 5E,F, the mRNA levels of PI3K and Akt were downregulated in the EPCs exposed to hypoxia compared with control cells (*P* < 0.05). ECSW treatment upregulated the mRNA levels of PI3K and Akt in EPCs exposed to hypoxia (*P* < 0.05). However, pretreatment with the PI3K inhibitor, LY294002, inhibited this effect of ECSW in EPCs exposed to hypoxia (*P* < 0.05). These data demonstrate that the EPCs injury induced by hypoxia and the protective effect of ECSW on EPCs injured by hypoxia may be attributed to the regulation of PI3K/Akt/eNOS signaling.

### ECSW Promote the Expression of Bcl-2, Increases NO Production, and Inhibits the Expression of Bax and Caspase3 in EPCs After Hypoxic Injury by Activating the PI3K/Akt/eNOS Signaling Pathways

**Figure 6:**
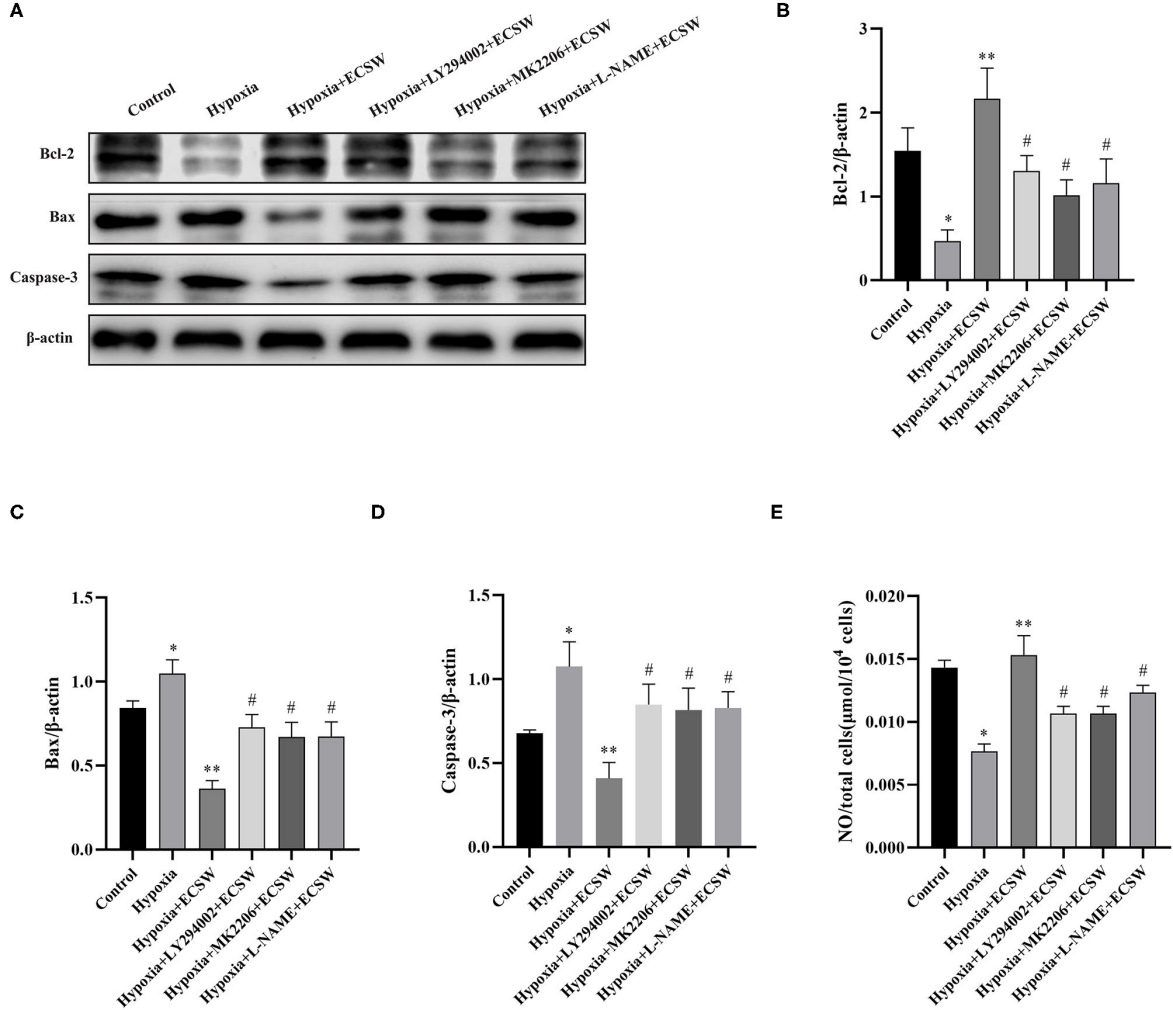
ECSW promote the expression of Bcl-2, increases NO Production, and inhibits the expression of Bax and Caspase3 in EPCs after hypoxic injury by activating PI3K/Akt/eNOS signaling pathway. **(A)** The expression of Bcl-2, Bax, Caspase3, and β-actin proteins of EPCs in each group. **(B–D)** Quantification analysis of Bcl-2, Bax, and Caspase3 expression levels, respectively. **(E)** Quantitative analysis of NO production of EPCs in each group. Data are presented as mean ± SD, *N* = 3. *P < 0.05 vs. group control, **P < 0.05 vs. group hypoxia, ^#^*P* < 0.05 vs. group hypoxia + ECSW.

Western blot assay was used to assess the expression of the downstream signaling molecules, Bcl-2, Bax and Caspase3, in the different groups. The results (Figures 6A–D) showed that the expression of Bcl-2 protein decreased (*P* < 0.05), but the expression of Bax and Caspase3 protein increased (*P* < 0.05) after exposing EPCs to hypoxic conditions for 24 h. After ECSW treatment, the expression of Bcl-2 protein significant increased (*P* < 0.05) and the expression of Bax and Caspase3 protein marked decreased in EPCs exposed to hypoxic conditions (*P* < 0.05). In addition, pretreatment with the PI3K inhibitor, LY294002, the Akt inhibitor, MK2206, and the eNOS inhibitor, L-NAME, inhibited the beneficial effects of ECSW in EPCs exposed to hypoxia (*P* < 0.05). As shown in Figure 6E, NO production was lower in the EPCs exposed to hypoxia compared with control cells (*P* < 0.05). Again, NO production in EPCs exposed to hypoxia improved after ECSW treatment (*P* < 0.05). In addition, pretreatment with the PI3K inhibitor, LY294002, the Akt inhibitor, MK2206, and the eNOS inhibitor, L-NAME, decreased NO production in EPCs exposed to hypoxia after ECSW treatment (*P* < 0.05).

## Discussion

The main pathological basis of (CHD) is the development of atherosclerosis, and the initial sign of atherosclerosis is damage to vascular endothelial cells (24, 25). In most cases, myocardial infarction occurs due to disruption of a vulnerable atherosclerotic plaque or erosion of the coronary artery endothelium. Following myocardial ischemia, increasing damage-associated insufficient oxygen and energy supply results in microvascular dysfunction and metabolic disorder, even cell death (26, 27). At the same time, it is accompanied by a series of parallel changes that include abnormal vascular wall tension balance associated with impaired nitric oxide synthesis and increased levels of angiotensin and endothelin to inhibit angiogenesis and vascular endothelial repair (28, 29). Importantly, previous studies have found that the number of circulating EPCs was obviously reduced in patients with CHD with attenuated function in neovascularization (30, 31), which were consistent with some related research on EPCs (32). Furthermore, lower levels and dysfunction of circulating EPCs were shown to be closely associated with poor prognosis in patients with CHD (33, 34) or animal models (35, 36).

To our best knowledge, the repair of damaged endothelium is of great worth in prevention and treatment of cardiovascular diseases. After sensing the damage to endothelial layer of arteries triggered by hypoxia injury, EPCs derived from the bone marrow or peripheral blood are recruited and home to sites of ischemia, then differentiate into mature endothelial cells to maintain the integrity of the vascular endothelium (37, 38). Therefore, EPCs as a group of stem cells with the potential of angiogenesis, play an essential role in endothelial repair. The overall increases in EPCs number and function have been proposed as an effective therapeutic means for CHD. In this study, we focused on the strategy for strengthening the functions of EPCs, and provided some basic evidence for extracorporeal cardiac shock waves (ECSW) therapy in CHD.

As a non-invasive physical stimulus, extracorporeal shock wave therapy has been widely used in clinical fields such as osteoarthritis (39), chronic pancreatitis (40), and renal calculus (41). In recent years, it has been found that ECSW can also promote angiogenesis, which not only provides a new idea for the treatment of CHD, but also provides a new choice for adjuvant therapy. Clinical studies have shown that ECSW treatment can improve the clinical symptoms and quality of life parameters of patients with CHD (8), but the exact mechanism of ECSW treatment has not yet been clarified. Previous studies have suggested that the main mechanism of ECSW therapy may be to bring about high expression of VEGF, eNOS, and other angiogenesis-related factors, resulting in enhanced mobilization of EPCs derived from autologous bone marrow to the damaged ischemic myocardium *in vivo* (21, 22, 42). However, the detailed mechanism in EPCs by which ECSW promotes angiogenesis remains unclear.

From the result of our previous exploratory experiment, ECSW has been found to result in satisfactory improvement the function of EPCs through activating of PI3K/Akt and MEK/ERK signaling pathways *in vitro* (43). However, above study has been limited by several experimental factors such as modeling condition and detailed pathway study. In the past, we did not consider the effect of hypoxic-ischemic microenvironment on EPCs while suffering CHD. ECSW was used only for EPCs in normoxic condition, which did not provide direct evidence for the application of ECSW for CHD. Therefore, in the present study, we firstly exposed EPCs to hypoxic conditions (1% O_2_, 95% N_2_, 5% CO_2_) and starved with 1% fetal bovine serum in EBM-2 medium for 24 h to simulate a hypoxic-ischemic injury. Besides, the EPCs were isolated from ApoE gene to knockout rat bone marrow for closer to the pathological model of atherosclerosis in CHD, which was better than before in study protocol.

The results in this study showed that dysfunction and apoptosis of EPCs were induced by hypoxia in line with previous studies (44, 45). Innovatively, ECSW can obviously promote the function of EPCs, especially angiogenesis, and inhibit the apoptosis of EPCs after hypoxic-ischemic injury. In order to elucidate the precise molecular mechanism of ECSW, we screened significant signaling pathways through bioinformatics data analysis. EPCs from the control, hypoxia, and hypoxia +ECSW groups were used for mRNA sequencing. KEGG enrichment analysis showed that differentially expressed genes were enriched in cancer signaling, PI3K-Akt signaling, and Rap1 signaling or other pathways (*P* < 0.01). What is different from our former experiment is that MEK/ERK signaling pathway was not statistically significant in this condition. We speculated probably a change of modeling condition. Another possible explanation for this might be weak activation of this pathway in ECSW ameliorating damaged EPCs, which may need further investigations. Inspired by the results of bioinformatics, we selected the differentially expressed genes related to PI3K-Akt signaling pathway and verified them in subsequent experiments. In great detail, PI3K inhibitor (LY294002), Akt inhibitor (MK-2206), and eNOS inhibitor (L-NAME) were used to block this signaling pathway when intervened by ECSW, showing a more complete mechanism study.

Here, we found that the hypoxia-induced EPCs dysfunction and apoptosis were consistent with the downregulation of p-PI3K, p-Akt, p-eNOS expression and decreased production of NO in EPCs, while ECSW treatment improved the function of EPCs after hypoxic injury with increased NO, p-PI3K, p-Akt, p-eNOS expression in EPCs. What's more, these pathway inhibitors impeded downstream signal in parallel with an elimination of ECSW effect on EPCs function. We observed that ECSW-mediated increase in these proteins in EPCs were reversed by pathway inhibitors, accompanied with an inhibitory effect on EPCs function including migratory, proliferative, adhesive, and tube formation capacities. These results indicated that the inhibition of PI3K/Akt/eNOS signaling pathway in EPCs may be a pathological mechanism for the reduction of endogenous vascular repair in CHD, but ECSW have shown promoting effects on EPCs function after hypoxic injury by activating in PI3K/Akt/eNOS signaling pathway.

PI3K/Akt is a classical signaling pathway that plays an important role in cell proliferation, migration, apoptosis, angiogenesis, and other biological processes. A few studies have shown that PI3K/Akt signaling pathway plays an important role in mobilizing and improving the function of EPCs, and its role is mainly based on the activation of the PI3K/Akt signaling pathway to induce downstream eNOS phosphorylation and nitric oxide production (46, 47), which were consistent with our findings. It has been noted that eNOS is potential for the regulation of EPC function, which can catalyze the production of NO from L-arginine, participating in regulating vascular homeostasis and arterial tone, also can promote angiogenesis in response to tissue ischemia (48, 49). Thus, eNOS and NO appear to be pivotal indicator of the function of EPCs. We found that ECSW have enhanced the expression of eNOS and promoted the production of NO in damaged EPCs, exhibiting a promising target for angiogenesis therapy in CHD.

The Bcl-2 protein family is a group of important apoptotic regulatory factors that controls the mitochondrial apoptosis pathway and plays an anti-apoptotic role (50, 51). Bax protein is found in the cytoplasm of cells and its structure is similar to that of Bcl-2, which contributes to cell apoptosis (52) under the stimulation of a series of apoptotic signals, Caspase-9 and Caspase-3 are activated to promote apoptosis. Bcl-2, Bax, and Caspase3, as downstream molecules, are involved in the regulation of cell apoptosis through the PI3K/Akt signaling pathway (53, 54). In this study, it was observed that ECSW upregulated the expression of Bcl-2 protein and inhibited the expression of Bax and Cleaved caspase-3 protein in EPCs after hypoxia. Together, these findings suggest that ECSW may improve hypoxia-induced EPCs apoptosis by regulating PI3K/Akt signaling pathway and its downstream molecules, which provide strong evidence for ECSW therapy in rescuing impaired EPCs.

There are several limitations that should be considered in the present study. Firstly, given that ECSW has been proven to bring effective benefits in CHD mostly by mobilizing EPCs in human or animal studies, so we provided in-depth research on the function and molecular mechanism of ECSW in EPCs. This study is limited *in vitro*, future investigation in animal models and even clinical trials is constantly needed. Secondly, based on the results of bioinformatics data, there are multiple signaling pathways possibly related to the effects of ECSW. We preliminarily verified the PI3K/Akt/eNOS signaling pathway and obtained satisfactory outcomes, but the contribution of other pathways should not be neglected and remain to be further explored. Thirdly, we used pathway inhibitors in this study, the PI3K/Akt/eNOS genes knockout models are needed for further verification.

## Conclusions

In conclusion, the findings of the present study provide valuable information. We found that the downregulation of PI3K/Akt/eNOS signaling pathway in EPCs were induced by hypoxic-ischemic injury with dysfunction and apoptosis of EPCs. ECSW can improve the function of EPCs including migratory, proliferative, adhesive, and tube formation capacities, and reduce the apoptosis of EPCs by activating PI3K/Akt/eNOS signaling pathway. After blocking this signaling pathway, the beneficial effects of ECSW on post-hypoxia EPCs were inhibited. Therefore, this work may provide new evidence for ECSW therapy in CHD through a potential mechanism on EPCs.

## Data Availability Statement

The datasets presented in this study can be found in online repositories. The names of the repository/repositories and accession number(s) can be found below: NCBI SRA BioProject, accession no: PRJNA752783.

## Ethics Statement

The animal study was reviewed and approved by The Institutional Animal Care and Use Committee (IACUC) of the Institutional Ethics Committee at the First Affiliated Hospital of Kunming Medical University.

## Author Contributions

HongyC designed and supervised the study. TG and HongbC supervised the study and critically revised the draft. XC and YM performed the statistical analyses and drafted the manuscript. MW, DY, ZH, and YS performed the experiments. All authors contributed to the article and approved the submitted version.

## Funding

This study was supported by the National Natural Science Foundation of China (No. 81760067), the Foundation Projects of Applied Basic Research Joint Science and Technology Ministry of Yunnan Province with Kunming Medical University (202001AY070001-027), Yunnan Province special Project for the Famous Medical Talents of the High-level-Talents Training Support (Grant No. 60120160407), Yunnan Province High-level Health Technical Talents (leading talents) (Grant No. L-2019025), the Construction Project of Clinical Medical Center of Cardiovascular and Cerebrovascular Disease of Yunnan Province (Grant No. ZX2019-03-01), the Scientific Research Fund Project of Education Department of Yunnan Province (2021Y339), and the Graduate Innovation Fund of Kunming Medical University (2021S031).

## Conflict of Interest

The authors declare that the research was conducted in the absence of any commercial or financial relationships that could be construed as a potential conflict of interest.

## Publisher's Note

All claims expressed in this article are solely those of the authors and do not necessarily represent those of their affiliated organizations, or those of the publisher, the editors and the reviewers. Any product that may be evaluated in this article, or claim that may be made by its manufacturer, is not guaranteed or endorsed by the publisher.

## Supplementary Material

Supplementary Table 1 EPCs_Control vs. EPCs_Hypoxia_GO_enrichment.xlsx.

Supplementary Table 2 EPCs_Hypoxia vs. EPCs_Hypoxia + ECSW_GO_enrichment.xlsx.

Supplementary Table 3 EPCs_Control vs. EPCs_Hypoxia_KEGG_enrichment.xlsx.

Supplementary Table 4 EPCs_Hypoxia vs. EPCs_Hypoxia + ECSW_KEGG_enrichment.xlsx.
