# Corrigendum: Extracorporeal Cardiac Shock Waves Therapy Improves the Function of Endothelial Progenitor Cells After Hypoxia Injury *via* Activating PI3K/Akt/eNOS Signal Pathway

**DOI:** 10.3389/fcvm.2021.793246

**Published:** 2021-12-22

**Authors:** Mingqiang Wang, Dan Yang, Zhao Hu, Yunke Shi, Yiming Ma, Xingyu Cao, Tao Guo, Hongbo Cai, Hongyan Cai

**Affiliations:** ^1^Department of Cardiology, The First Affiliated Hospital of Kunming Medical University, Kunming, China; ^2^Department of Cardiology, Yunnan Fuwai Cardiovascular Hospital, Kunming, China; ^3^Department of Vascular Surgery, The First Affiliated Hospital of Kunming Medical University, Kunming, China

**Keywords:** extracorporeal cardiac shock waves, endothelial progenitor cells, cell function, PI3K/Akt/eNOS signaling pathways, hypoxia injury, nitric oxide (NO)

In the original article, there was a mistake in Figure 4 as published. Because of our carelessness in combining images, we put the wrong images on Figures 4C and E. The corrected Figure 4 appears below.

**Figure 4:**
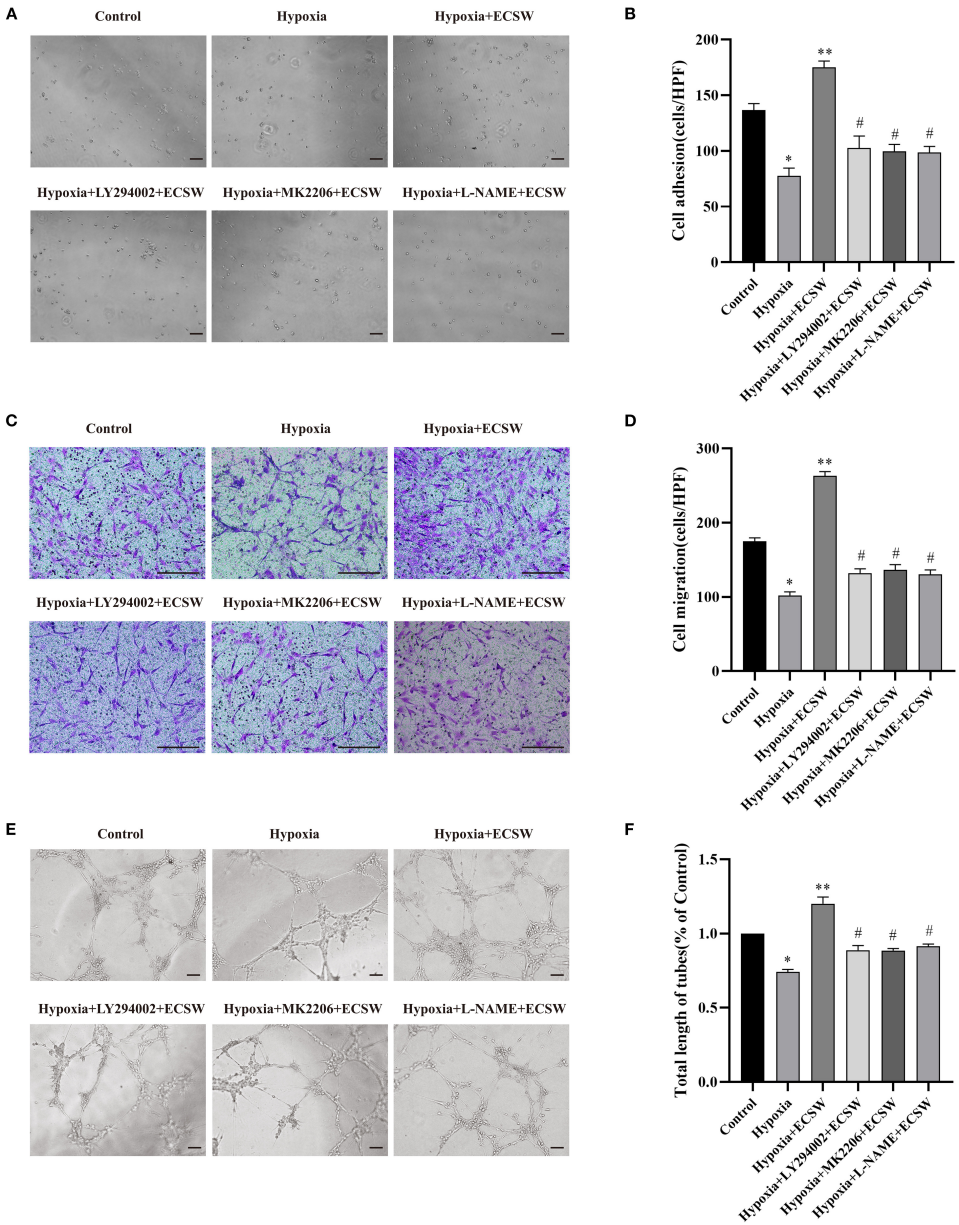
ECSW improve the adhesive, migratory, and tube formation capacity of EPCs after hypoxic injury by activating PI3K/Akt/eNOS signaling pathway. **(A,C,E)** Representative images of EPCs adhesive, migratory, and tube formation in each group under a microscope. **(A,E)** Scale bar = 100 μm; **(C)** Scale bar = 200 μm. **(B,D,F)** Quantitative analysis of the adhesive, migratory, and tube formation of EPCs in each group. Data are presented as mean ± SD, *N* = 3. *P < 0.05 vs. group control, **P < 0.05 vs. group hypoxia, #*P* < 0.05 vs. group hypoxia + ECSW.

The authors apologize for this error and state that this does not change the scientific conclusions of the article in any way. The original article has been updated.

## Publisher's Note

All claims expressed in this article are solely those of the authors and do not necessarily represent those of their affiliated organizations, or those of the publisher, the editors and the reviewers. Any product that may be evaluated in this article, or claim that may be made by its manufacturer, is not guaranteed or endorsed by the publisher.

